# Endometrioid Adenocarcinoma in the Native Ureter of a Renal Transplant Patient: Case Report and Review of the Literature

**DOI:** 10.1100/tsw.2010.166

**Published:** 2010-09-01

**Authors:** Kelly A. Healy, Kenneth J. Carney, Adeboye O. Osunkoya

**Affiliations:** ^1^ Department of Urology Emory, University School of Medicine, Atlanta, GA, USA; ^2^ Department of Pathology Emory, University School of Medicine, Atlanta, GA, USA

**Keywords:** endometriosis, adenocarcinoma, urinary tract, hormonal replacement therapy

## Abstract

Endometriosis is characterized by endometrial-like tissue outside the uterus, primarily on the pelvic peritoneum, ovaries, and rectovaginal septum, and, in rare cases, within the urinary tract (1–3%). Although endometriosis is a benign condition, malignant transformation of endometriosis is a well-described phenomenon. Malignancies arising in endometriosis are uncommon at extragonadal pelvic sites. A case of endometrioid adenocarcinoma in the native ureter of a postmenopausal renal transplant patient presented with painless gross hematuria and hydroureteronephrosis. The patient had a history of total abdominal hysterectomy and bilateral salpingo-oophrectomy 14 years prior for menorrhagia and had since been on unopposed estrogen replacement therapy. Workup revealed a filling defect in the native left mid-ureter secondary to a large 2.5-cm ureteral tumor. Endoscopic biopsies of the native left ureteral mass showed endometrioid adenocarcinoma, grade II-III. The patient ultimately underwent an open native left nephroureterectomy and temporary diverting colostomy. Final pathology confirmed endometrioid adenocarcinoma, grade II-III, arising in a background of endometriosis with negative perirectal lymph nodes. This case of ureteral endometrioid adenocarcinoma highlights the importance of obtaining a careful history and maintaining a high index of suspicion for malignant degeneration, especially in the context of hyperestrogenism.

## INTRODUCTION

While endometriosis is a benign disease, malignant transformation may occur in up to 1% of women[1]. The majority of cases (80%) arises in ovarian endometriosis, whereas the remaining 20% occurs in extragonadal sites[2]. Limited studies have reported on malignant tumors originating from genitourinary endometriosis. Herein, we describe a case of ureteral endometrioid adenocarcinoma in the native ureter of a renal transplant patient.

## CASE PRESENTATION

The patient is a 51-year-old female, lifetime nonsmoker, with a history of end-stage renal disease secondary to glomerular nephritis status post failed initial transplant. She subsequently underwent explantation followed by left-sided renal transplant 30 years ago. She presented for evaluation of new-onset painless gross hematuria. Her urologic history was otherwise unremarkable. She denied any history of urolithiasis, urinary tract infections (UTIs), dysuria, frequency, or urgency; no associated weight loss, fatigue, or change in bowel habits. She did note dyspareunia and was status post total abdominal hysterectomy and bilateral salpingo-oopherectomy (TAH and BSO) due to menorrhagia at the age of 37. At that time, she was started on unopposed estrogen replacement therapy, which she had continued for the past 14 years. Initial evaluation for her gross hematuria was completed at an outside institution. Physician examination was unremarkable. Laboratory studies were normal, including a negative urine culture and cytology. Her serum creatinine (Cr) was normal at a baseline of 0.8 mg/dL. Computed tomography (CT) scan of the abdomen and pelvis revealed expected findings of severely atrophic native kidneys bilaterally with thinned parenchyma (Figs. 1 and 2). However, a filling defect was also seen in the native left mid-ureter over the sacroiliac joint with proximal left hydroureteronephrosis, suspicious for urothelial carcinoma. Additionally, CT showed a large periureteral soft tissue mass, measuring 5.0 cm in greatest dimension, encasing the mid- to distal native left ureter. Cystoscopy showed normal bladder mucosa. Retrograde pyelography confirmed preoperative CT findings. On ureteroscopy, a large approximate 2.5-cm soft tissue mass was visualized within the native left mid-ureter. This was reportedly not a typical papillary-appearing tumor, but rather polypoid. Ureteroscopic biopsies of the tumor revealed endometriosis. Further review by a separate pathologist supported this diagnosis. More specifically, the biopsy showed the presence of cilia and stromal components, and lack of high-grade cytology. Based on these findings, the final pathology was deemed benign and most consistent with endometriosis with hypoplasia.

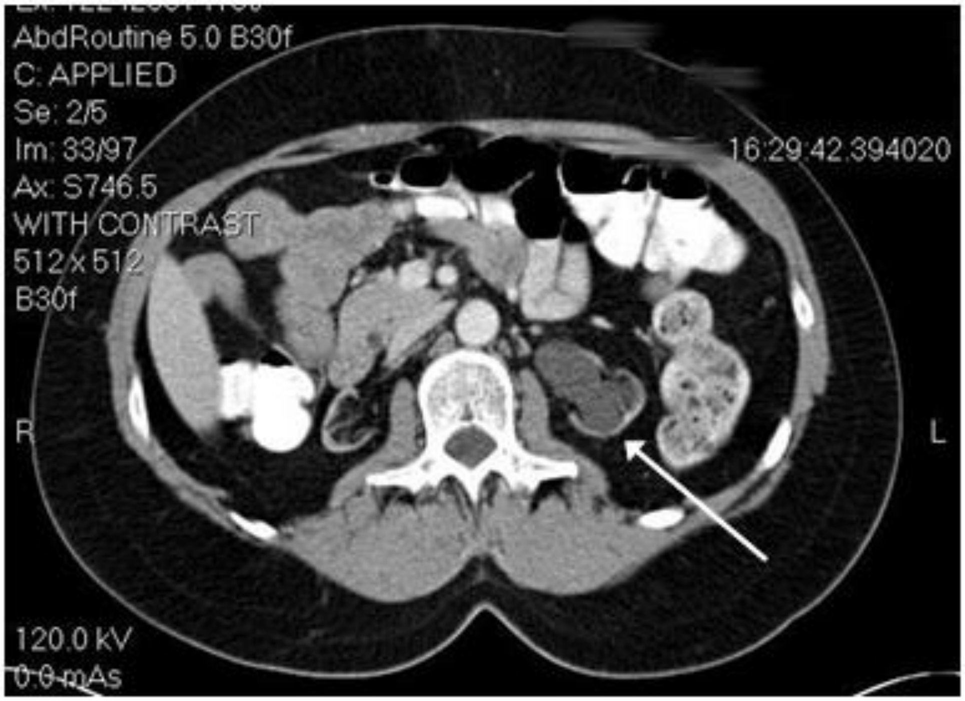

**FIGURE 1.** Initial CT of abdomen and pelvis demonstrated severely atrophic bilateral native kidneys with severe left-sided hydroureteronephrosis.

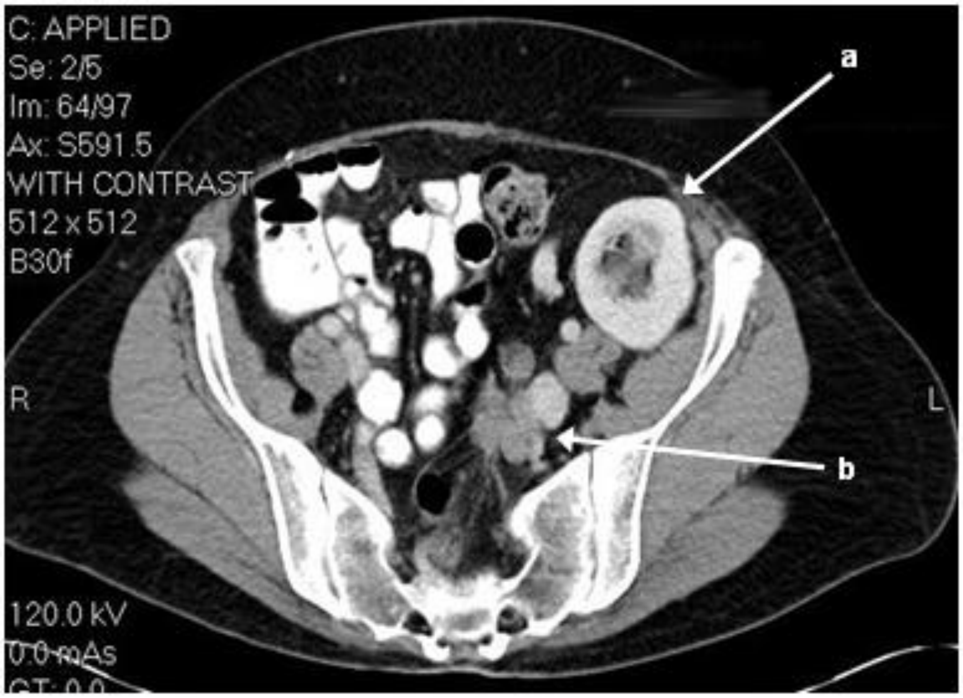

**FIGURE 2**. CT of abdomen and pelvis also showed a normal-appearing left transplant kidney (a) and a filling defect in the native left mid-ureter over the sacroiliac joint, with an associated large 5-cm periureteral soft tissue mass (b).

The patient was then referred to our tertiary care university hospital for further management. Given the unusual nature of this lesion, she underwent repeat cystoscopy, ureteroscopy, and ureteroscopic biopsy. However, retrograde biopsy of this ureteral mass proved difficult. The decision was made for an antegrade percutaneous approach in order to obtain adequate tissue specimen. Following percutaneous nephrostomy tube placement, she underwent cystoscopy, antegrade renoscopy and ureteroscopy, biopsy of ureteral mass, stent placement, and nephrostomy tube change. Approximately 70% of the entirety of the mass was removed using a tipless nitinol basket via a flexible cystoscope. Indeed, the ureteral mass appeared smooth and lacked characteristic papillary features. Final pathology of the native left ureteral mass revealed endometrioid adenocarcinoma, grade II-III (Fig. 3).

In preparation for definitive resection, repeat CT scan was performed and confirmed the initial radiographic findings. A large, lobulated enhancing soft tissue mass (5.0 × 2.5 × 4.1 cm) was seen intimately associated with the native left mid- to distal ureter. Mild mass effect was visible on the adjacent left iliac vein, but without evidence of invasion. No pelvic or retroperitoneal adenopathy was evident. Chest X-ray showed no pulmonary metastases. The large tumor size precluded an endoscopic approach. Therefore, the patient was taken to the operating room for an open left native nephroureterectomy. Preoperatively, the nephrostomy tube was removed, but the indwelling ureteral stent was left in place to facilitate intraoperative identification of the left native ureter. Intraoperatively, the location of the tumor below the transplanted kidney and significant adhesions from prior pelvic surgeries made exposure rather difficult. Access to the left native ureter also required mobilization of the left transplant kidney. The large mass-like lesion was easily identifiable and, on its lateral aspect, adherent to the renal vein of the transplant kidney. With the assistance of the vascular and transplant surgeons, the main vein from the transplant kidney was sacrificed in order to remove the tumor in its entirety. Venous outflow was restored using a left saphenous vein bypass from the transplant renal vein to the left common iliac vein. Moreover, the tumor invaded the mesentery of the rectum and encased the inferior mesenteric artery. This significant tumor burden necessitated removal of rectal mesentery, division of the inferior mesenteric artery, proctectomy, and diverting colostomy.

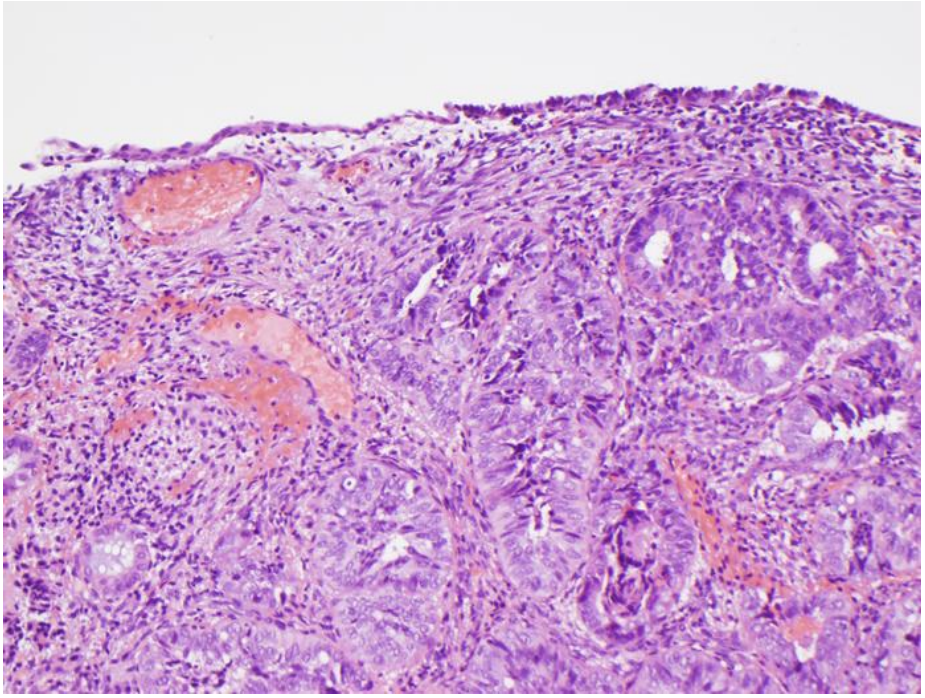

**FIGURE 3.** Section of ureter with endometrioid adenocarcinoma undermining a thin strip of benign urothelium.

Postoperatively, her hospital course was prolonged and complicated by acute respiratory distress syndrome, nonoliguric renal failure with a peak Cr of 4.7 mg/dL, and left common femoral vein deep vein thrombosis (DVT). She was ultimately discharged home on postoperative day 16 with a Cr of 1.6 mg/dL. She was continued on anticoagulation medication for 6 months for her DVT. At last follow-up, her Cr was improved to 0.8 mg/dL. She has been evaluated by Gynecologic Oncology and is scheduled to start adjuvant chemotherapy with carboplatin and Taxol.

## PATHOLOGIC FINDINGS

Final surgical pathology of the left native ureteral tumor revealed endometrioid adenocarcinoma, grade II-III, arising in a background of endometriosis (Figs. 4–6). The tumor, which measured 2.5 × 1.5 cm, invaded through the ureteric wall and extended into the ureteral lumen. Additionally, multiple foci of endometriosis were present within the perirectal adipose tissue similar to those identified in the periureteric tissue in association with the endometrioid adenocarcinoma. Seven perirectal lymph nodes showed reactive changes, but no evidence of malignancy.

## DISCUSSION

Endometriosis is characterized by the growth of endometrial glands and stroma outside of the myometrium or uterine cavity. This benign disease is quite common and affects approximately 15% of the premenopausal female population[3]. Patients typically present with pelvic pain and subfertility. While the exact etiology is unknown, the most widely accepted theories on the pathogenesis include (1) retrograde menstruation through the fallopian tubes, with pelvic spillage and implantation; and (2) dissemination through the lymphatic channels or blood vessels[4]. Ectopic endometrial tissue is hormonally

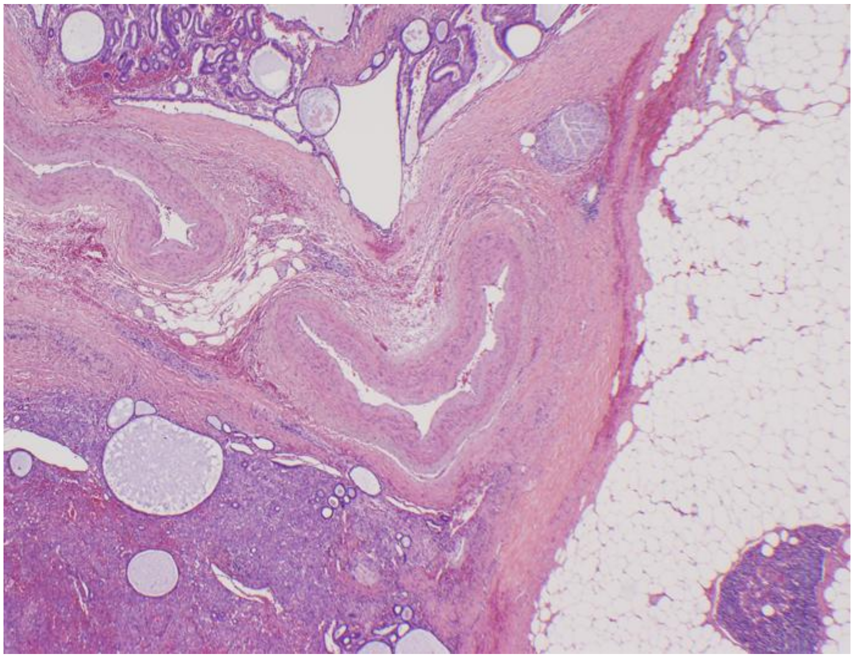

**FIGURE 4.** Section of ureter with endometrioid adenocarcinoma arising in a background of endometriosis (lower left corner).

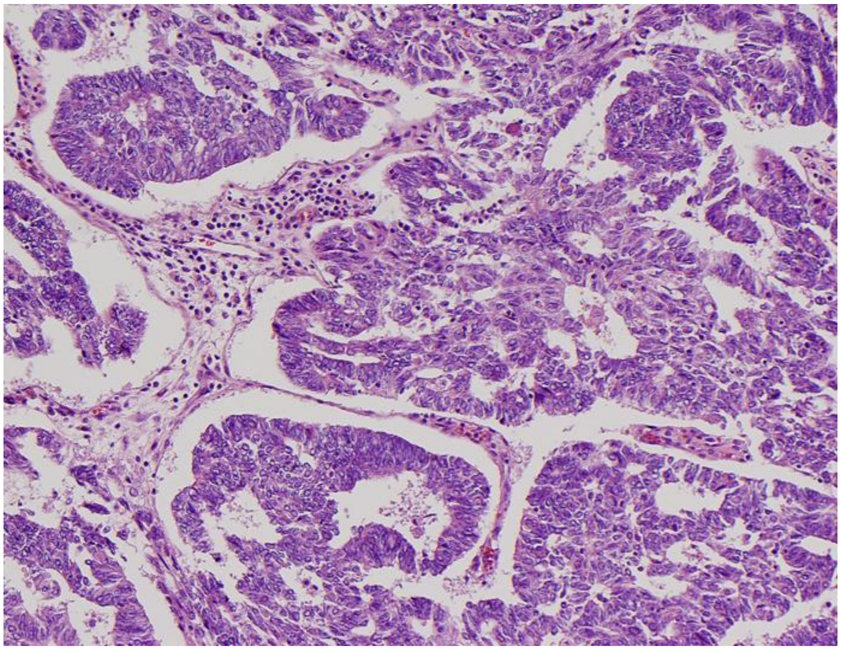

**FIGURE 5.** Section of ureter with endometrioid adenocarcinoma forming glandular structures.

responsive and undergoes proliferation and secretion similar to the uterine lining[1,4]. In other words, it is incapable of autonomous growth and thus not neoplastic. Albeit benign, endometriosis may demonstrate aggressive pathology marked by high local invasiveness and high recurrence[5,6]. Furthermore, malignant transformation may develop and the existence of malignances arising in endometriosis has been appreciated for quite some time[7].

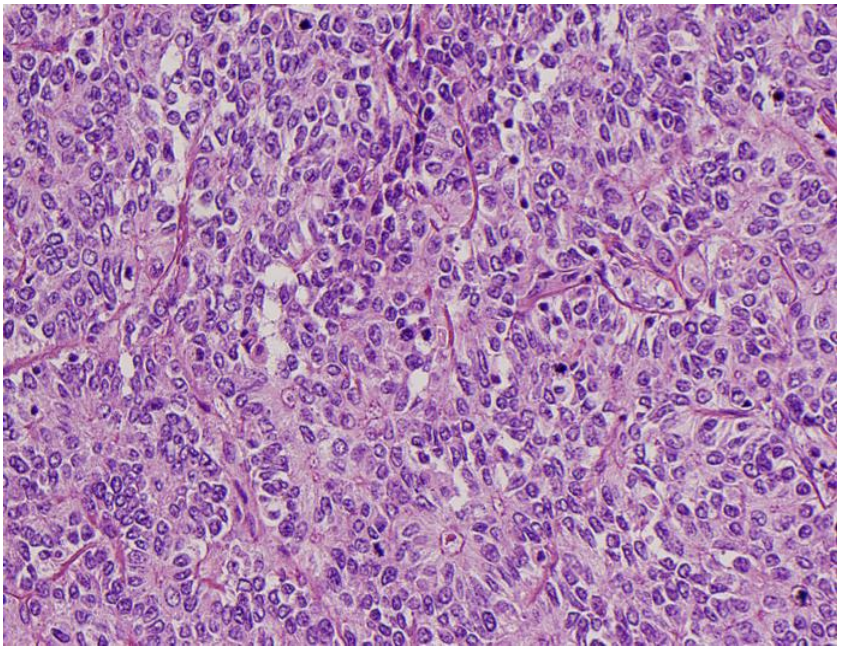

**FIGURE 6.** Section of ureter with endometrioid adenocarcinoma forming solid sheets.

Although endometriosis occurs most frequently in the pelvic organs, it may also occur outside the normal confines of the reproductive tract. Genitourinary involvement is exceedingly rare with an incidence of 1–3% of all cases of endometriosis. The bladder, ureter, and kidney are affected in a ratio of 40:5:1[5,6]. Most affected women are of premenopausal age (25–40 years), yet the disease may occur after menopause[8]. The majority of postmenopausal cases occur in women with high serum levels of estrogen[6,9,10].

Hyperestrogenism is either endogenous due to obesity or exogenous due to hormonal replacement therapy (HRT). Since endometriosis is a hormone-dependent disease, hyperestrogenism may induce hyperplasia or malignant degeneration of the ectopic endometrial tissue. In particular, hyperestrogenism secondary to unopposed estrogen therapy has been implicated as a risk factor in the development of malignancy[2]. Zanetta et al. conducted a 10-year retrospective review to characterize the clinical and pathologic features of malignancies developing from endometriosis (n = 31)[10]. Taken together, obesity and unopposed estrogen therapy were significantly associated with the malignant transformation of endometriosis. The median duration of unopposed estrogen therapy was 10 years (range: 2–20). Several authors advocate the addition of progestins to HRT in women with a history of endometriosis in order to decrease the risk of malignant transformation[7]. Congruent with these findings, our patient reported a history of unopposed estrogen HRT for 14 years after a TAH and BSO. Following the initial pathological findings, this was discontinued and she was started on venlafaxine to control her postmenopausal symptoms of hot flashes.

In addition to hyperestrogenism, prior surgical manipulation is a recognized risk factor for the development of urinary tract endometriosis. This is thought to pose an increased risk due to iatrogenic implantation of endometriotic foci[3,4]. Several series have noted a high incidence of prior pelvic surgery between 60–70% in patients with endometriosis of the urinary tract.

Ureteral endometriosis accounts for only a minority of cases (0.1–0.4%)[5]. Endometriosis of the ureter is categorized as either intrinsic (with glands and stroma within the ureteral lamina propria, tunica muscularis, or lumen) or extrinsic (with external compression around the adventitia from adjacent disease in the ovary, broad ligament, or uterosacral ligament). Extrinsic disease is four times more common than intrinsic disease[2,3,4]. Whereas intrinsic ureteral disease likely arises from lymphovascular metastases, extrinsic disease may originate from intraperitoneal seeding of regurgitated endometrial cells or from compression by extensive involvement of the adjacent pelvic organs[2]. Both types of ureteral endometriosis may result in ureteral obstruction[8]. Ureteral endometriosis almost exclusively occurs below the pelvic brim in the lower third of the ureter[4], as in our patient. Unilateral involvement is more common than bilateral disease. Interestingly, ureteral endometriosis is more common on the left side, compatible with the menstrual reflux theory and anatomic difference in the right and left hemipelvis[2,5]. That is, the presence of the sigmoid colon on the left is thought to delay the spread of retrograde menstrual blood. Our case confirms this finding, underscoring involvement of the left side in 70% of unilateral cases.

Although an established relationship exists between cystitis cystica and bladder endometriosis, ureteral endometriosis is not associated with ureteritis cystica[6]. Oftentimes, patients present with nonspecific symptoms, making preoperative diagnosis difficult[5]. Symptoms may be secondary to the pelvic endometriosis itself and include dysmenorrhea, menorrhagia, irregular menses, or pelvic pain[4,6]. Far less commonly, symptoms may be due to urinary tract involvement. In fact, flank pain, fever, chills, dysuria, frequency, and UTI occur in less than 25% of patients. Intrinsic endometriosis may be associated with lateralized pathognomonic gross hematuria synchronized with the menstrual cycle, as the ectopic endometrial tissue responds to hormone stimulation[5]. However, clinical symptoms are often silent and accounted for 30.8% of patients in one series. Unfortunately, a large proportion of patients present with silent obstructive uropathy or occasionally renal failure[6].

Malignant transformation of endometriosis is a well-recognized phenomenon, with over 300 cases reported in the literature since the 1920s. The exact incidence is difficult to ascertain, but this may occur in 1–2% of cases of endometriosis[2,7]. First introduced by Sampson in 1925, histologic criteria used for establishing the malignant transformation in endometriosis include (1) presence of benign endometriosis, (2) endometriosis in close proximity to malignancy, and (3) malignant tissue histology[1,11]. Scott later modified these criteria in 1953 to include the histologic transition from benign to atypical to malignant tissue[7]. Histologic subtypes of malignancies arising in endometriosis parallel the normal histologic components of endometriosis. Epithelial cell types most commonly include endometrioid adenocarcinoma, followed by clear cell carcinoma, and, rarely, serous carcinoma[1,7,12]. Stromal components may also undergo malignant transformation and lead to stromal sarcomas. Finally, mixed cell types with both epithelial and stromal elements have been described[7].

While the majority of these cases originates from ovarian endometriosis, approximately 20–25% occur in extragonadal sites, including the genitourinary system[2,3,7]. Most patients with malignant degeneration into endometrioid carcinoma present with signs and symptoms analogous to ovarian or primary peritoneal malignancies, including bloating or abdominal distension, increased abdominal girth, or change in bowel habits[7]. The gold standard for diagnosis remains definitive histologic evaluation using Sampson’s criteria. Treatment depends on a multimodality approach. First, surgical excision is performed to both establish a diagnosis and, if possible, completely resect the disease. Depending on histology and disease spread, adjuvant treatment may consist of chemotherapy, radiation therapy, and/or hormonal therapy[7,13]. Chemotherapy regimens typically consist of the combination of paclitaxel with one of the platinum agents. Similar to endometrial cancer, adjuvant therapy may include radiation therapy either after surgery, or for treatment of local recurrence after primary surgery and chemotherapy. However, in our patient with a history of renal transplant, radiation therapy was not a feasible option. Finally, progestational agents have been employed as anticancer agents in endometrial cancer. Endometrioid adenocarcinomas arising in endometriosis demonstrate analogous histology and, therefore, these agents may be utilized for such patients. Progestational agents are indicated for patients with tumor recurrence despite initial chemotherapy and/or radiation therapy, and patients with medical comorbidities that preclude other therapies[7].

## CONCLUSIONS

Genitourinary endometriosis is an atypical manifestation of a benign and common disease. Although rare, malignant transformation of endometriosis is a well-known phenomenon. While the majority of cases arise in ovarian endometriosis, up to 25% may occur in extragonadal sites. This interesting case of ureteral endometrioid adenocarcinoma highlights the importance of maintaining a high index of suspicion in females who present for urological evaluation, particularly in the setting of hyperestrogenism.
